# Talus Fracture Dislocation Management With Crossed Kirschner Wires in Children

**DOI:** 10.7759/cureus.13801

**Published:** 2021-03-10

**Authors:** Gur Aziz Singh Sidhu, Jamie Hind, Neil Ashwood, Harjot Kaur, Andrew Lacon

**Affiliations:** 1 Trauma and Orthopaedics, University Hospitals of Derby and Burton NHS Foundation Trust, Burton-on-Trent, GBR; 2 Anesthesia, Dayanand Medical College and Hospital, Ludhiana, IND

**Keywords:** talus fracture dislocation, pediatric fractures, foot fractures, kirschner wires

## Abstract

Skeletal trauma accounts for 10% to 15% of all childhood injuries, with approximately 15% to 30% of these representing physeal injuries. Talus fractures are rare injuries in children with an estimated prevalence of 0.008% of all childhood fractures. Cast immobilization is sufficient treatment for non-displaced fractures, however displaced fractures of the talus require surgical intervention to minimize the risk of trauma-related avascular necrosis (AVN) due to disruption of the vascular supply originating from the talar neck. A 13-year-old boy was brought to the accident and emergency (A/E) department following a road traffic accident while he was pillion riding a bike. Following the accident, he was unable to bear weight on his right foot and his anterior ankle region was swollen, with no neurological deficit or open wound. He had no other injury and no medical or surgical history. On review of the ankle and foot radiographs, he was noted to have a right talar neck fracture with subtalar and ankle dislocation. His computer tomographic (CT) images demonstrated a Hawkins Type IV talus fracture.

Initial treatment involved a plaster of Paris (POP) back slab with the ankle in a neutral position. His right leg was elevated on pillows and treated with elevation and ice to alleviate the swelling. As the fracture was comminuted and displaced with ankle and subtalar dislocation, operative intervention (open reduction and fixation of talus with crossed K wires) was planned. The patient was discharged in below knee slab which was changed to a non-walking cast at two weeks. The patient was kept non-weight bearing until fracture united. These types of fractures are rare in children and proper clinical and radiological evaluation is essential. Such fractures should be reduced as early as possible to reduce the ischemia time thus prevent the chances of osteonecrosis. Lastly avoid tourniquets and stable anatomical reduction of fracture is must.

## Introduction

Around 10% to 15% of all childhood injuries are caused by skeletal trauma, with approximately 15% to 30% of these are physeal injuries (phalanx fractures are the most common physeal injury) [1]. Increased sports participation of children in recent years has been attributed to the increased incidence of fractures. The peak incidence of fractures in boys occurs at age 16 years (450 per 10,000 per year) and in girls occurs at age 12 years (250 per 10,000 per year) [1]. Talus fractures are rare injuries in children with an estimated prevalence of 0.008% of all childhood fractures [2].

The pediatric talus can sustain higher forces as compared to adults because the pediatric foot is flexible and has higher elastic resistance than adult bone [3,4]. The most common fracture site is the talar neck, followed by talar body. Cast immobilization is sufficient treatment for non-displaced fractures; however displaced fractures of the talus require surgical intervention to minimize the risk of trauma-related avascular necrosis (AVN) due to disruption of the vascular supply originating from the talar neck.

## Case presentation

**Figure 1:**
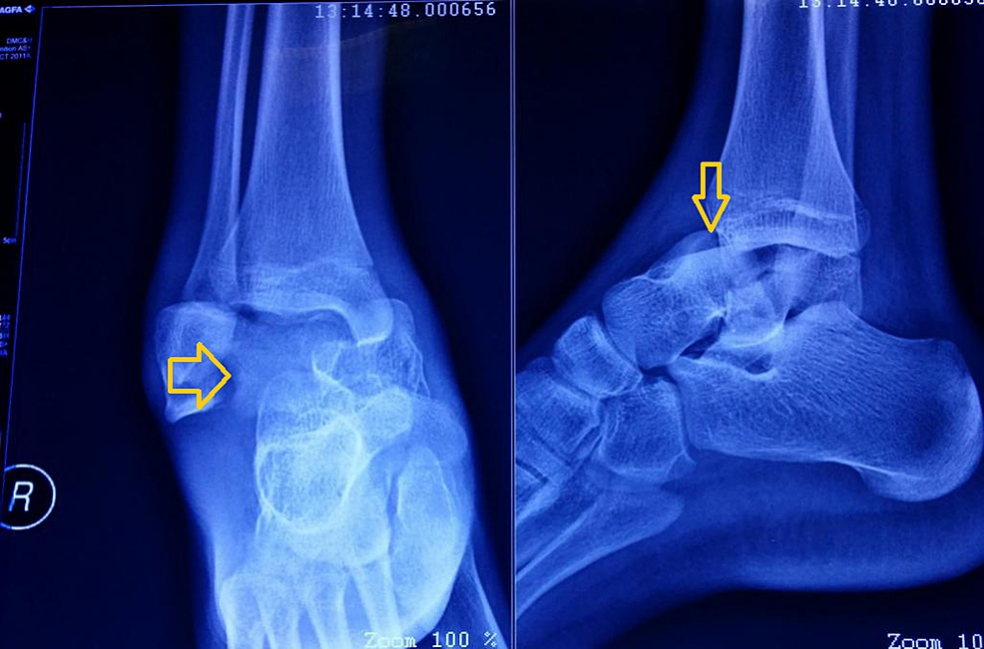
Anteroposterior and lateral radiographs of right ankle

A 13-year-old boy was brought to the emergency department following a road traffic accident while he was pillion riding a bike. Following the accident, he was unable to bear weight on his right foot and his anterior ankle region was swollen, with no neurological deficit or open wound. He had no other injury and no medical or surgical history. Following triage, he was sent for radiographs of ankle (Figure 1). Splinting was done in form of above knee plaster of Paris (POP) slab along with elevation over pillows and ice packs.

On review of the ankle and foot radiographs, he was noted to have a right talar neck fracture with subtalar and ankle dislocation (Figure 1).

**Figure 2:**
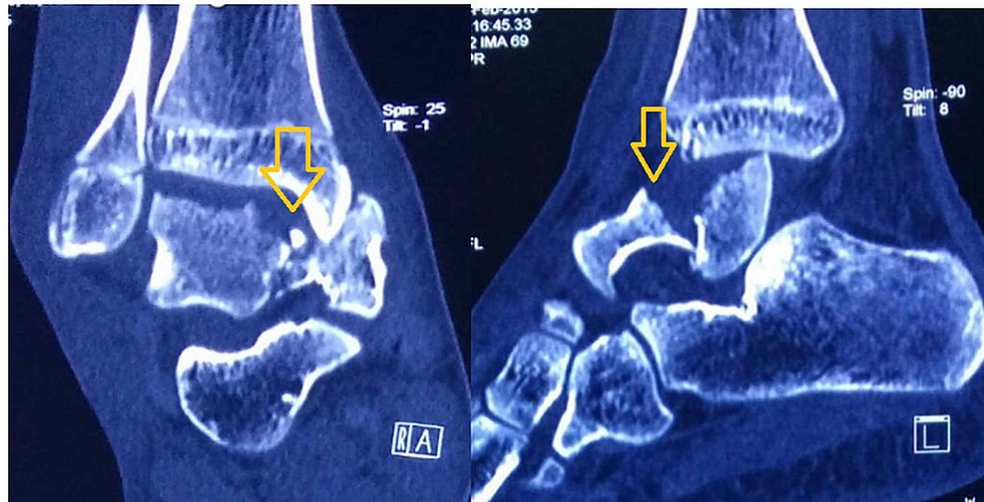
Computer tomography scan images demonstrating fracture

CT scan was done to delineate fracture pattern and plan for surgery. The CT scan images demonstrated a Hawkins Type IV talus fracture with dislocation (Figure 2).

**Figure 3:**
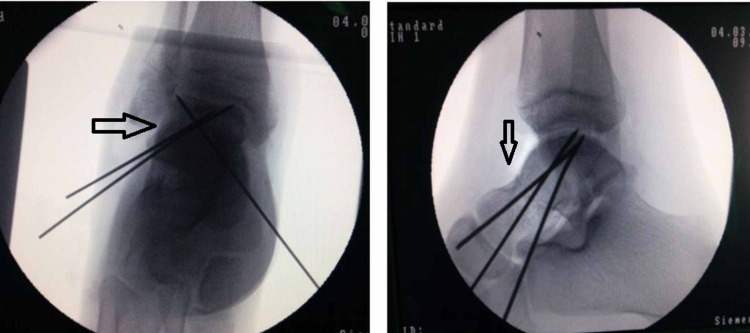
Intraoperative pictures under image intensifier

After initial treatment with back slab, elevation and ice packs, the decision for closed/open reduction and fixation was taken. Ankle was approached with dual incision and fracture was reduced. The reduction was checked under image intensifier and found satisfactory (Figure 3).

Stabilization of fracture was done using crossed Kirschner wires which were buried in the skin and sutures applied. A below knee back slab was given initially and the patient was kept non-weight bearing.

**Figure 4:**
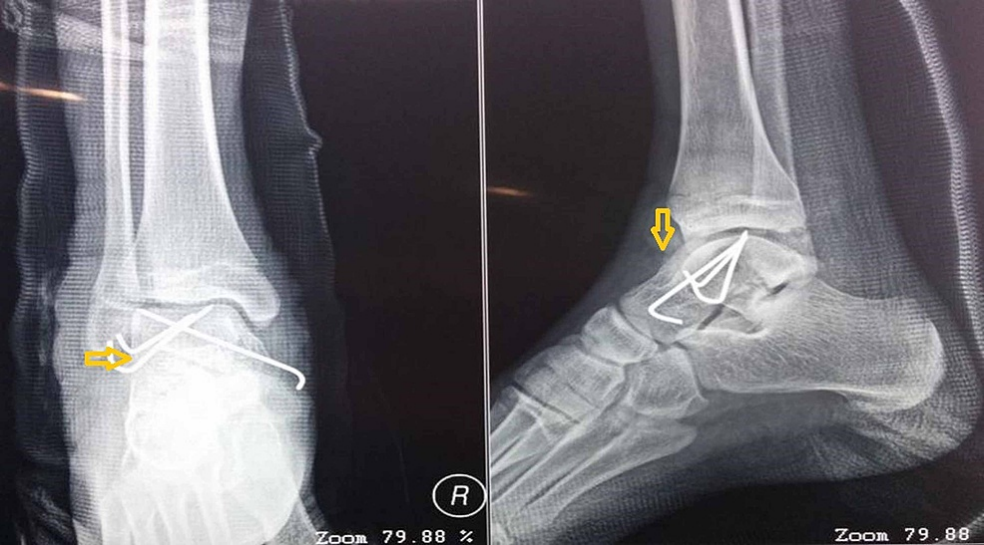
Post operative follow-up radiographs

Sutures were removed on 14th post operative day and the patient was given below knee cast and kept non-weight bearing for another six weeks (Figure 4).

The patient was on regular follow-up clinically and radiographically to assess for avascular necrosis of the talus. The fracture united around three months and K wires were removed.

## Discussion

Aviator’s astragalus was first described by Anderson resulting from dorsiflexion mechanism of injury [5]. The most common cause for pediatric talus fractures is road traffic accidents followed by fall from height [4,6]. Displaced talar neck fractures carry a worse prognosis both in children as well as adults [4].

Few classifications systems have been mentioned in literature [7,8]. Delee gave a five-part classification for talar fractures: type 1, transchondral dome fractures; type 2, shear fractures; type 3, posterior tubercle fractures; type 4, lateral process fractures; and type 5, crush fractures [7]. Hawkins classification system most commonly used included four types: type I, non-displaced fracture; type II, displaced fracture with subluxation or dislocation of the subtalar joint; type III, displaced fracture with body of talus dislocated from both subtalar and ankle joint; type IV, type III with dislocation or subluxation of the head of the talus at the talonavicular joint [8]. Our case was type Hawkins type IV. One important point to remember was a displaced talar neck was a prerequisite for Hawkins classification system [8].

A study conducted by Smith et al. reported that displaced pediatric talar fractures and those associated with high energy trauma resulted in more complications like arthrosis, avascular necrosis, delayed union, neuropraxia, etc. Moreover, such fractures occurred with more severity in older boys [9]. Moreover, it was mentioned that no persistent osteonecrosis was observed in patients younger than 12 years, and favourable outcomes have been reported in literature irrespective of the mode of treatment [4]. Excellent long-term prognosis of minimally displaced and undisplaced fractures of the talus in the pediatric population was reported by Jensen et al. [3].

Complications like avascular necrosis, pain and degenerative arthritis were seen more often after open reduction and internal fixation of talar fractures. This might be postulated due to longer expectancy of children and adequate fracture reduction [10]. Anatomic reduction and stable fixation with minimal soft tissue injury of displaced fractures is of utmost importance to reduce these complications.

Review of literature reported conflicting data on osteonecrosis in pediatric talar fractures. Letts and Gibeault reported a 25% incidence of osteonecrosis in their study of 12 patients [11]. Two of these three patients had non-displaced fractures which were undiagnosed initially. Similarly, 27% incidence of AVN was reported by Linhart and Hollwarth [12]. On contrary, Jensen et al. reported no AVN changes in 11 non-displaced and three displaced talar fractures in children [3].

Few studies reported that the use of tourniquet can cause osteonecrosis of talus [13,14]. When the talus fractures, the vessels become disrupted and the reperfusion does not occur until reduction of the fracture. Longer the time taken for reduction more is the ischemic time and more are chances of osteonecrosis [3]. Moreover, the application of a tourniquet, which lengthens ischemic time, can therefore be a negative factor for blood supply and can contribute to subsequent osteonecrosis [13].

Although literature reported few case reports regarding such fractures in children but these could be often missed [15]. Proper clinical and radiological evaluation is essential. Such fractures should be reduced as early as possible to reduce the ischemia time thus prevent the chances of osteonecrosis. Lastly, avoid tourniquets and stable anatomical reduction of fracture is must.

## Conclusions

Talus fractures are rare injuries in childhood and could often be missed. These are complex injuries requiring immediate closed/open reduction and fixation by either K wires or screws to reduce ischemia time. Anatomic reduction of the fracture and stabilization is a must to prevent complications including avascular necrosis of talus.
